# Core competencies for UK occupational health nurses: a Delphi study

**DOI:** 10.1093/occmed/kqw089

**Published:** 2016-08-04

**Authors:** D. Lalloo, E. Demou, S. Kiran, M. Gaffney, M. Stevenson, E. B. Macdonald

**Affiliations:** ^1^Healthy Working Lives Group, Institute of Health and Wellbeing, College of Medical, Veterinary and Life Sciences, University of Glasgow, Glasgow G12 8RZ, UK,; ^2^MRC/CSO Social and Public Health Sciences Unit, Institute of Health and Wellbeing, College of Medical, Veterinary and Life Sciences, University of Glasgow, Glasgow G2 3QB, UK,; ^3^Department of Occupational Health and Medicine, Institute of Public Health, Hacettepe University, Sihhiye-Ankara 06100, Turkey,; ^4^Specialist Community Public Health Nursing (Occupational Health), University of the West Of Scotland, Paisley Campus, Glasgow PA1 2BE, UK.

**Keywords:** Competencies, Delphi study, occupational health nursing, occupational health nurse training.

## Abstract

**Background:**

Occupational health nurses (OHNs) play a pivotal role in the delivery of occupational health (OH) services. Specific competency guidance has been developed in a number of countries, including the UK. While it is acknowledged that UK OHN practice has evolved in recent years, there has been no formal research to capture these developments to ensure that training and curricula remain up-to-date and reflect current practice.

**Aims:**

To identify current priorities among UK OHNs of the competencies required for OH practice.

**Methods:**

A modified Delphi study undertaken among representative OHN networks in the UK. This formed part of a larger study including UK and international occupational physicians. The study was conducted in two rounds using a questionnaire based on available guidance on training competencies for OH practice, the published literature, expert panel reviews and conference discussions.

**Results:**

Consensus among OHNs was high with 7 out of the 12 domains scoring 100% in rating. ‘Good clinical care’ was the principal domain ranked most important, followed by ‘general principles of assessment & management of occupational hazards to health’. ‘Research methods’ and ‘teaching & educational supervision’ were considered least important.

**Conclusions:**

This study has established UK OHNs’ current priorities on the competencies required for OH practice. The timing of this paper is opportune with the formal launch of the Faculty of Occupational Health Nursing planned in 2018 and should inform the development of competency requirements as part of the Faculty’s goals for standard setting in OHN education and training.

## Introduction

Occupational health nurses (OHNs) play a key role in occupational health (OH) services globally [1] and are the single largest healthcare profession involved in workplace health management in Europe [2]. Working at the interface between the workforce and management and as the first contact point for many health-related problems, they need to be able to respond to a wide range of health issues and questions [3], and require skills to meet the workforce’s physical and psychosocial needs [4,5]. The scope of OH nursing practice is broad [1,2,4–7], although to some degree context specific to individual workplaces and requirements [1,8]. OHNs can have varying roles including clinician/practitioner, educator, manager/administrator and consultant [1] with functions within these roles expanding due to economic, sociopolitical and technological demands. Nursing bodies including the World Health Organization (WHO)—European Member States, American Association of Occupational Health Nurses (AAOHN), Australian College of Occupational Health Nurses (ACOHN), and the UK Royal College of Nursing (RCN), have defined key OHN functions as guidance and a foundation of practice [2,9–11], leading to the development of specific core competencies. In some countries, these are based on levels of achievement (competent, proficient/experienced, expert) [9]; in others, they are classified into ‘traditional role’ and ‘emergent role’ activities [10] with substantial overlap in the competencies required.

UK OH nursing is highly developed, comprising both registered and specialist nurses, the latter undertaking additional formal OH studies. A competency framework for OHNs was published by the RCN UK in 2011 to provide a benchmark in planning developments for practice [11]. The original 2004 publication used competencies identified through consultation with UK OHNs and allied professions. Currently, there are ~3400 OHNs on the Nursing and Midwifery Council (NMC) register [6] and there may be other non-OH qualified nurses working in the field, to whom these competencies would apply. The first OHN consultant post was established in 2002 and a Faculty of Occupational Health Nursing (FOHN) is currently being established to provide a unified voice for the profession [12]. Its vision is ‘to promote excellence in the education, research and evidence-based practice of all OHNs for the benefit of the working population’ [13].

OH practice has changed in the 140 years since the first ‘industrial nurse’ was appointed. The initial focus on prevention and management of occupational disease and injury has shifted to encompass areas including health promotion and maintaining work ability and well-being. Routine tasks are increasingly undertaken by OH technicians. Concurrently, OHN roles have developed with many OHNs leading services and controlling resources and budgets [6]. OH practice is constantly evolving and OHNs have to develop continuously and adapt competencies based upon core skills, resulting in OH nursing becoming a mix of ‘traditional’ and ‘emergent’ functions [14]. OHN roles and competencies have been the subject of research [15–23]. Two Australian studies [20,23] identified that OHNs were not engaging fully in all practice areas defined in their competency standards. More traditional roles (e.g. treatment and health assessment) were deemed more applicable to their practice than emergent activities (e.g. health promotion, education and training) and a substantial amount of time was spent on them. Managing an OH service was the only emergent activity identified as a substantial part of the OHN’s role. A lack of involvement in research was highlighted [20] and another study related this to a lack of skills in, and knowledge of, research methods [16]. In a global survey of OH professionals [15], clinically focused competencies were the skills most frequently considered necessary for OHNs, with lower scores for research. Employers’ expectations as a factor determining OHN activities have also been studied [17–19]. The OHN competencies most valued by employers were effective communication, understanding the relationship between occupational exposures and health outcomes and staying current in one’s field of practice [17]. Competencies receiving the lowest scores were all related to research [17].

Our literature review did not identify any recent study on core competency requirements for UK OHNs. Our study aimed to identify and obtain consensus on current competency priorities for UK OHNs. The FOHN formal launch is planned in 2018 and an objective is for it to be central to OHN standard setting for education, training and practice supervision [12]. Phase 2, to be launched in 2016, is to include a research project to gather information about the profession and the building of the FOHN. The timing of this study is therefore opportune in informing competency requirements as part of the Faculty’s goals for standard setting, and for the development of specialist educational courses in OH nursing.

## Methods

**Figure 1.**
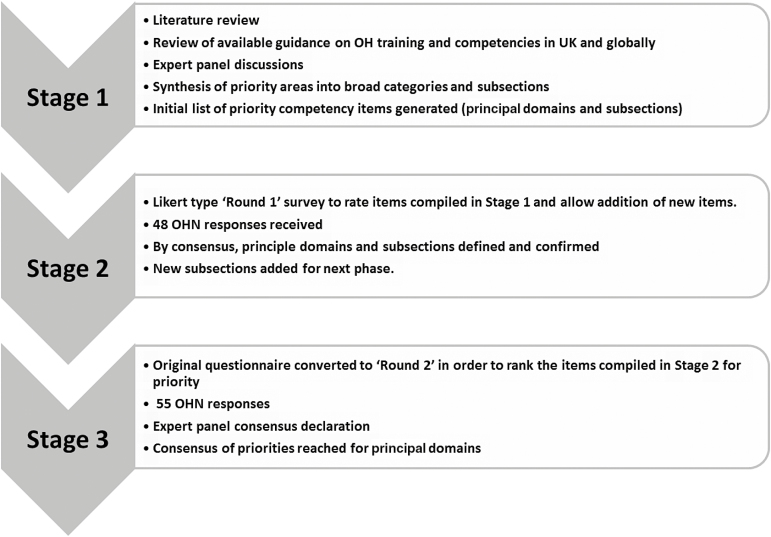
The modified Delphi process described.

We conducted a modified Delphi study among UK OHNs described in Figure 1. A detailed description of the methodology used is presented elsewhere, as this formed part of a larger Delphi study [24]. In stage 1 (questionnaire development), we undertook a literature review and reviewed available definitions [17] and guidance on training competencies for OH practice. For the purpose of this study, we defined competencies as ‘a group of required knowledge, skills and abilities for safe and effective OH nursing practice’. Expert panel discussions on competencies and the available guidance took place face-to-face and by e-mail. These included senior UK OHNs, international occupational physicians (OPs) and the author team. Key competency headings, defined here as ‘principal domains’, and specific topic areas within these (‘subsections’) were identified and agreed by the panel. We developed an initial questionnaire based on these discussions and training/competency guidance for OH practitioners from a range of sources including the nursing bodies AAOHN, ACOHN and RCN as well as WHO (European Member States) and the occupational medicine specialist training syllabus of a number of countries (including USA, Australia and the UK). We also included emerging areas of practice identified from the literature and within the speciality, notably the ageing worker [25], sickness absence management [26] and vocational rehabilitation [27].

We established contacts with key members from representative UK OH nursing networks who agreed to participate and disseminate the questionnaire. These included:

- JISCM@IL online discussion group for OH practitioners
- RCN nurses mailing list
- Members of the Association of OH Nurse Practitioners (AOHNP)
- Association of OH Nurse Managers Group
- Association of OH Nurse Educators (Jisc)
- University of the West of Scotland (UWS) mailing list of past students and practice teachers
- National Health Service (NHS) OH nurses Scotland
- The Society of Occupational Medicine

We carried out the survey in two rounds (Figure 1). In stage 2 (round 1 ‘rating’ questionnaire), the first questionnaire comprised 12 principal domains and within each domain were subsections detailing specific domain competencies. This ranged from one principal domain with three subsections to another with 11. Respondents were asked to indicate whether or not they considered the principal domain to be important and then to rate the relative importance of the subsection items on a score from 0 to 5 (0 indicated that the item was ‘not necessary’ and 5 that it was ‘most important or essential’). We included open-ended questions, allowing respondents to add to the lists. The preliminary results from round 1 were presented at an international conference on occupational medicine training competencies (The European Association of the Schools of Occupational Medicine 2014, Glasgow). All new competency items suggested were from round 1 and these discussions informed the development of the second questionnaire.

In stage 3 (round 2 ‘ranking’ questionnaire), once round 1 responses were collated and evaluated, we produced a second modified questionnaire. This retained the same 12 principal domains but included additional subsection items derived from the first round open-ended question responses and conference discussions. No original items were removed. This second questionnaire was circulated to the same key contacts as round 1 for distribution. OHNs were invited to participate irrespective of whether they had taken part in the first round or not. In round 2, we asked respondents to rank the principal domains and the subsections. The item most important was ranked 1, next most important 2 and so on. Respondents were not permitted to give two items the same score. A section for comments was included, although additional items were not invited in this round. The questionnaire is available in the Data, available as Supplementary data at *Occupational Medicine* Online. We analysed responses by averaging the rank orders to produce a mean score for each principal domain and subsection item. As domains had different numbers of subsections, mean scores were standardized to a 1–10 scale, to allow comparison of the relative importance of subsection items between domains. The standardized mean score gives an indication of the consensus of opinion. The subsection mean standardized scores were subsequently weighted using a scale from 1–12 based on the ranking order of their respective principal domains to allow inter-domain comparisons.

Questionnaires were circulated in English using a SMART survey link via electronic mail and piloted in advance by a representative sample of seven OHNs. A participant information sheet was included and consent was required before completing the questionnaire. The University of Glasgow, College of Medical, Veterinary & Life Sciences Ethics Committee provided ethics approval (200130150). Round 1 took place between June and August 2014 and round 2 between January and April 2015. Reminder emails were sent ~1 month after questionnaire distribution. Analysis was performed using SPSS Statistics v21 [28]. For both rounds, we reviewed respondents’ job titles and place of work and classified them into three main job categories. If exclusively involved in clinical OH practice, they were categorized as ‘OHN’; if they had a management title, they were labelled ‘OHN/Manager’ and if they undertook an academic role, they were classified as ‘OHN/Academic’. Categories of OH practice comprised work in a (healthcare) setting, e.g. hospital, public/private sector organization (industry), teaching or research (academic) or any work sector not covered by these (other). Respondents self-selected their sector type and could choose more than one category.

## Results

Round 1 (rating): 48 responses were received and respondents’ demographics are presented in Table 1. The majority 96% (46) were female and 81% (39) were aged 45–64. The distribution by job category was 73% (35) OHN and 27% (13) OHN/manager. Mean years of experience was 17.6±8.4 (min = 1; max = 38). The main area of practice was healthcare (48%), followed by industry (44%) and academia (4%) although a degree of crossover occurred. Respondents regarded all competency areas as important (Table 2), with scores of 87% and above in every domain (no statistically significant difference between domains). Seven principal domains scored 100% (Table 2). The new subsection items derived from the open-ended questions from this round included the principles of toxicology, ergonomics, occupational/industrial hygiene and travel medicine (within ‘General principles of assessment & management of occupational hazards to health’), motivational interviewing (within ‘Communication skills’) and additional environmental health competencies.

**Table 1.** Responses by age, sex, job title, practice area and years of experience for rounds 1 and 2

Features	Round 1 (*n* = 48), *n* (%)	Round 2 (*n* = 55), *n* (%)
Age range category		
25–34	3 (6)	1 (2)
35–44	6 (12)	4 (7)
45–54	24 (50)	32 (59)
55–64	15 (31)	16 (30)
65–74	–	1 (2)
Total	48 (100)	54 (100)
Sex		
Male	2 (4)	3 (6)
Female	46 (96)	50 (94)
Total	48 (100)	53 (100)
Job title		
OHN/manager	13 (27)	29 (53)
OHN	35 (73)	26 (47)
Total	48 (100)	55 (100)
Practice area		
Health care	23 (48)	20 (36)
Industry	21 (44)	28 (51)
Academic	2 (4)	8 (14)
Other	5 (10)	5 (9)
Total^a^	51	61
Years of experience	Mean ± SD (min–max)	Mean ± SD (min–max)
	17.6±8.4 (1–38)	19.4±8.3 (1–41)

^a^Total >48 because three respondents worked in more than one practice area.

**Table 2.** Priorities in principal domains (rating round 1 results)

Principal domains – rating round	Yes (%)	No (%)	Not relevant (%)
General principles of assessment and management of occupational hazards to health	100	–	–
Assessment of disability and fitness for work	100	–	–
Health promotion	100	–	–
Ethical and legal issues	100	–	–
Clinical governance/clinical improvement	100	–	–
Communication skills	100	–	–
Team working and leadership skills	100	–	–
Management skills	98	–	2
Environmental issues related to work practice	98	2	–
Teaching and educational supervision	94	6	–
Good clinical care	92	4	4
Research methods	87	13	–

Round 2 (ranking): 55 responses were received (Table 1). Distribution by job category was 47% OHN and 53% OHN/manager with a mean 19.4±8.3 years of experience. The main area of practice was industry (51%), followed by healthcare services (36%) and academia (14%) with a degree of crossover. Among participants in round 2, 16% (9/55) had participated in round 1 and 82% (45/55) only participated in round 2. A statistically significant difference between respondents of both rounds was demonstrated only for job title (Fisher exact test, *P* < 0.01). In round 2, ‘good clinical care’ was the principal domain ranked most important, followed by ‘general principles of assessment & management of occupational hazards to health and communication skills’ (Table 3). ‘Research methods’ was the domain considered least important, followed by ‘teaching & educational supervision’ and ‘health promotion’. The top scoring subsections by principal domain are also presented in Table 3 (all subsection ranks can be accessed in the Data, available as Supplementary data at *Occupational Medicine* Online). Within all domains the highest scoring subsections reflect what could be considered the ‘core’ activities within those domains, including taking an appropriate clinical/occupational history and understanding and applying the principles of risk assessment. Although not competencies per se, the free comments highlighted ‘awareness of one’s own capabilities and limitations’ and ‘seeking more expert advice or advice from another member of the OH team, when required’, as important.

**Table 3.** Principal domain rankings and top scoring subsections within each domain

Rank^a^	Ranked principal domains	Mean, rank ± SD	Weighted standard rank for subsection
	Highest ranked subsection within each domain		
1	Good clinical care	2.6±2.5	
	Take and analyse a clinical and occupational history, including an exposure history in a relevant, succinct and systematic manner	2.4±2.0	4.1
2	General principles of assessment and management of occupational hazards to health	3.2±2.3	
	Understand and apply the principles of risk assessment, i.e. recognition of potential hazards in the work environment, evaluating risks and providing advice and information on control measures	2.2±1.9	4.2
3	Communication skills	4.2±2.7	
	Be able to communicate effectively both orally and in writing to patients and other stakeholders in a manner that they understand	1.4±0.6	4.2
4	Assessment of disability and fitness for work	4.6±2.1	
	Assessing and advising on impairment, disability and fitness for work	1.6±0.9	6.4
5	Ethical and legal issues	5.8±2.1	
	Be well informed about acts, regulations, codes of practice and guidance relevant to the workplace setting	2.1±1.2	17.6
6	Clinical governance/clinical improvement	5.8±2.6	
	Practise evidence-based medicine	1.4±0.9	8.4
7	Team working and leadership skills	6.4±2.6	
	Understand how a team works effectively	2.2±1.4	21.9
8	Environmental issues related to work practice	7.4±2.1	
	Recognize and advise on health risks in the general environment arising from industrial activities	1.7±1.2	18.3
9	Management skills	8.3±3.1	
	Be able to understand the principles and practice of management	2.5±1.6	26.6
10	Health promotion	8.7±2.4	
	Assessing needs for health promotion in a workforce	1.3±0.5	25.3
11	Teaching and educational supervision	9.9±1.6	
	Identify learning outcomes and construct educational objectives	2.1±1.6	24.9
12	Research methods	11.3±1.2	
	Be able to define a problem in terms of needs for an evidence base	1.9±1.3	25.5

^a^Low scores indicate that many respondents gave this item high priority and high scores that they gave an item low priority.

## Discussion

This study established current competency priorities for OH practice among UK OHNs. OHN consensus was very high with seven of the 12 identified domains scoring 100% in rating. Although ‘good clinical care’ did not have 100% consensus in rating, it was ultimately ranked the most important domain. This discordance may reflect differences in interpretation when simply described as a domain, with improved understanding once more detailed subsections were presented. The top 5 ranked domains support a more traditional ‘clinically focused’ view of required competencies, as other competencies such as research and teaching ranked lower. In both rounds, ‘research methods’ was considered least important. No additional comments were provided to clarify the reasons for this. From the literature, lack of skills and knowledge in research methods [16], employer expectations and their perception of research as a ‘less valuable’ competency have been highlighted as key determining factors [17–19]. ‘Health promotion’ was ranked third lowest, despite an increasing focus on employee well-being and the pivotal role of OHNs in this. The comments section provided explanations that while OHNs considered health promotion important, lack of time and resources means that in practice higher priority roles such as health surveillance take precedent.

Our findings could reflect UK current service provision models which largely fall into two categories:

1. In-house services, employed directly by the organization and providing services to employees but often forming part of the organization’s decision-making structure and hence having opportunities to influence organizational culture and strategic direction. In addition, if the service is OHN led, there may be the requirement for management, teaching, leadership and research to be part of the OHN role.
2. Externally contracted services. These may be delivered at ‘arms-length’, where there is more of a customer relationship with the organization and employees are service consumers. In this case, OHN competencies required will be dictated by the customer and more likely sit in the traditional established areas described in ‘good clinical care’.

In-house service OHNs may develop high levels of competence that can be role- and employment sector-specific, e.g. in the nuclear industry and health sectors. Externally contracted OHNs, employed in more generic roles and often providing services over a range of organizations and geographical areas, may not have opportunities to be closely involved with an organization and in such settings research activities are not generally a primary function. The comments provided support this, emphasizing that for a number of domains, notably ‘clinical governance/improvement’, ‘teaching & educational supervision’ and ‘management’, importance varies by sector and area of practice. They also highlight that certain domains may be more applicable to more advanced, senior or experienced practitioners than, for example, to a newly qualified OHN. Although ‘research methods’ was ranked lowest overall as a principal domain, ‘practising evidence-based medicine’ was the top ranked subsection within ‘clinical governance/clinical improvement’. This may indicate that while involvement in research and in-depth knowledge of research methods is of less priority, the importance of an evidence base in clinical practice is acknowledged.

To our knowledge, this is the first formal UK study to identify priorities among OHNs for the competencies required for OH practice. A strength is that consensus was derived from OHNs working in a range of sectors and including managers and academics. UK OH nursing is continuously evolving with more routine tasks being transferred to technicians and OHN roles being enhanced [29], with many OHNs leading services and undertaking similar clinical activities to OPs, e.g. management referrals. A further strength of this study is capturing these current developments, so training and curricula remain up-to-date and reflective of current practice. Potential confounders were mitigated by not presenting the domains in any particular order to avoid bias, piloting both questionnaires for comprehension and ease-of-use, using an expert panel in the study development, inviting participants to add domains/subsections for missed competencies in round 1 and permitting participation in round 2 irrespective of round 1 participation. The low response rate can be considered a study limitation and it is possible that these results may not be representative of all UK OHNs. Other Internet-based surveys and competency studies have also demonstrated low response rates [15,30]. From an OHN perspective, there have been recent concerns about a lack of engagement and direction within the discipline [6], which may explain this response rate. It has been argued that the profession is currently under-represented at national level with only a minority of OHNs belonging to a dedicated OH organization [6]. A goal in the establishment of the FOHN is to address this.

Our findings are consistent with previous studies [15,17,20,23], with ‘good clinical care’ and ‘communication skills’ being high priorities and ‘research’ a lower priority. In a global survey of OH professionals for OHNs, ‘clinically focused’ competencies were the skills most frequently noted. Scores for research were low relative to other OH professionals and few countries identified basic research as part of a nurse’s expected role [15]. In two Australian studies [20,23], OHNs saw ‘traditional role’ (clinically based) activities as more applicable to their practice rather than ‘emergent role’ activities such as health promotion, education and training and research. Our findings also highlight some correlation between OHN and employer perspectives with effective communication also high on employers’ priority lists and research considered least important [17]. This study has identified that respondents harbour more traditional ‘clinically focussed’ views of required competencies. This may reflect the fact that traditional activities currently dominate OHN work and/or differences in OH service provision models.

This study represents an important step in informing competency priorities and requirements among UK OHNs. This is especially timely with the establishment of the FOHN, where project leaders have identified standard setting for education and training as key priorities. These findings can be used to inform discussions within the profession, as well as larger studies with representative OHN samples and potentially involving different stakeholders (e.g. employers and trade unions). Opportunities for further research could include exploration of the drivers of traditional and emergent roles and in-depth investigation of which competencies apply at different levels of seniority and experience.

**Key points**

- This study represents an important step in informing competency priorities and requirements for UK occupational health nurses, with the finding that respondents harbour more traditional ‘clinically focused’ views of required competencies.
- This may reflect that traditional activities currently dominate occupational health nurse work and/or differences in occupational health service provision models.
- With the formal launch of the Faculty of Occupational Health Nursing planned in 2018, the timing of this paper is opportune, enabling it to inform a key faculty goal for standard setting in education and training.

## Funding

Medical Research Council (partnership grant MC/PC/13027) to E.D.; Hacettepe University (international collaboration grant BAP ID:5080) to S.K.

## Conflicts of interest

None declared.

## Supplementary Material

Supplementary Data
