# Supplementary material for: Core competencies for UK occupational health nurses: a Delphi study
Source: Occup Med (Lond). 2016 Aug 4;66(8):649–55. doi: 10.1093/occmed/kqw089 (PMC5088609; doi:10.1093/occmed/kqw089)
Supplement: Supplementary Data [file supp_66_8_649__index.html]

Core competencies for UK occupational health nurses: a Delphi study — Supplementary Data 

# Core competencies for UK occupational health nurses: a Delphi study

## Supplementary Data

Data files

- Supplementary Data - Supplementary Data
