# Supplementary material for: Core competencies for UK occupational health nurses: a Delphi study
Source: Occup Med (Lond). 2016 Aug 4;66(8):649–55. doi: 10.1093/occmed/kqw089 (PMC5088609; doi:10.1093/occmed/kqw089)
Supplement: Supplementary Data [file supp_kqw089_Lalloo_et_al_Supplementary_data_OM_02_05_16.docx]

**Core competencies for occupational health nurses in the UK: A modified Delphi study**

Drushca Lalloo^1,^*, Evangelia Demou^1,2^, Sibel Kiran^3^, Mairi Gaffney ^1^, Marisa Stevenson^4^ Ewan. B. Macdonald^1^.

**[Supplementary data]**

1. Healthy Working Lives Group, Institute of Health and Wellbeing, College of Medical, Veterinary and Life Sciences, University of Glasgow, G12 8RZ, Glasgow, UK
2. MRC/CSO Social and Public Health Sciences Unit, Institute of Health and Wellbeing, College of Medical, Veterinary and Life Sciences, University of Glasgow, G2 3QB, Glasgow, UK
3. Hacettepe University, Institute of Public Health, Department Occupational Health and Medicine, Sihhiye-Ankara, Turkey
4. University of The West Of Scotland, Paisley Campus, PA1 2BE, Glasgow, UK

***Corresponding Author:**

Dr Drushca Lalloo

Healthy Working Lives Group, Institute of Health and Wellbeing

College of Medical, Veterinary and Life Sciences

University of Glasgow

1 Lilybank Gardens

G12 8RZ, Glasgow, UK

Email: [drushca@hotmail.com](mailto:drushca@hotmail.com)

Tel: 0141 3303559

Table S1. Ranking (Delphi-Round 2) Questionnaire for Occupational Health Nurses [distributed electronically using SmartSurvey]

STUDY OF THE COMPETENCIES OF OCCUPATIONAL HEALTH NURSES (OHN's) WITHIN THE UK-ROUND 2-RANKING PHASE

**By completing this questionnaire, you are consenting to take part in this entirely voluntary study**

**1. Please tick to confirm you have read the above statement ***

|  | Yes |
| --- | --- |

**You may receive this questionnaire several times- please only complete once.**

**2. Job title:***

|  |
| --- |

**3. Country of practice: ***

|  |
| --- |

**4. Gender:**

|  | Male |
| --- | --- |
|  | Female |

**5. Age range:**

|  | 18-24yrs |
| --- | --- |
|  | 25-34yrs |
|  | 35-44 yrs |
|  | 45-54 yrs |
|  | 55-64 yrs |
|  | 65-74 yrs |

**6. Years of experience in specialty:**

|  |
| --- |

**7. Which professional specialty body are you registered with or board certified by?**

|  |
| --- |

**8. Main area of current OH practise:**

|  | Healthcare |
| --- | --- |
|  | Industry |
|  | Academic |
|  | Other |

Specify

|  |
| --- |

**9. Did you complete a questionnaire in the first round of this study?
(please note, participation in the first round is not a requirement to take part in this questionnaire)**

|  | Yes |
| --- | --- |

|  | No |
| --- | --- |

**The following is a list of the elements Occupational Health Nurses are expected to be competent in when they complete their training. They comprise principle domains and then subsections. How important are they to you?**

**Please RANK them in order of importance using the scale ranges described at the start of each section. *No two items can be awarded the same score.***

***Please note: Once you rank the first (and each subsequent item), the subsections will auto-adjust to list your rank choices in chronological order. This will enable you to check and review your choices before proceeding to the next domain.***

**A Good Clinical Care**

**Please RANK each subsection in terms of their importance;**

**1= of most importance to 9 = of least importance***

| Broad general medical knowledge of all common medical conditions including diagnostic criteria, evidence based treatment spectrum and prognosis | \|  \| \| --- \| |
| --- | --- | --- |
| Take and analyse a clinical and occupational history including an exposure history in a relevant, succint and systematic manner | \|  \| \| --- \| |
| Perform a reliable and appropriate examination | \|  \| \| --- \| |
| Interpret the results of investigations, including especially those relating to occupational attribution and functional prognosis | \|  \| \| --- \| |
| Perform investigations competently where relevant | \|  \| \| --- \| |
| Record concisely, accurately, confidentially and legibly all medical records, and date and sign all records | \|  \| \| --- \| |
| Show empathy with and listen to employee. | \|  \| \| --- \| |
| Appreciate the importance and interaction of psychological and social factors in patient’s disease and illness behaviour. | \|  \| \| --- \| |
| Manage time and problems effectively. | \|  \| \| --- \| |

**B General principles of assessment & management of occupational hazards to health**

**Please RANK each subsection in terms of their importance;**

**1= of most importance to 11 = of least importance***

| Understand and apply the principles of risk assessment- ie recognition of potential hazards in the work environment, evaluating risks and providing advice and information on control measures. | \|  \| \| --- \| |
| --- | --- | --- |
| Provide advice on medical aspects/ factors relevant to the risk assessment. | \|  \| \| --- \| |
| Carry out a workplace visit and produce a report. | \|  \| \| --- \| |
| Understand the core principles of occupational/ industrial hygiene and be able to interpret reports and related measurements. | \|  \| \| --- \| |
| Understand the core principles of ergonomics and be able to interpret reports. | \|  \| \| --- \| |
| Understand the core principles of toxicology and be able to interpret reports and related measurements. | \|  \| \| --- \| |
| Identify work related ill health and provide advice on prognosis, prevention and management. | \|  \| \| --- \| |
| Carry out and evaluate health surveillance including biological monitoring for workers exposed to occupational hazards. | \|  \| \| --- \| |
| Evaluate and advise on first aid facilities in the workplace. | \|  \| \| --- \| |
| Advising on disaster and contingency planning within the organisation. | \|  \| \| --- \| |
| Understand the principles of Travel Medicine | \|  \| \| --- \| |

**C  Assessment of disability and fitness for work.**

**Please RANK each subsection in terms of their importance;**

**1= of most importance to 10 = of least importance***

| Assessing and advising on impairment, disability and fitness for work | \|  \| \| --- \| |
| --- | --- | --- |
| Advising on rehabilitation | \|  \| \| --- \| |
| Advising on redeployment | \|  \| \| --- \| |
| Assessing and advising on early retirement due to ill-health | \|  \| \| --- \| |
| Assessing and advising on sickness absence | \|  \| \| --- \| |
| Advising on legal issues related to disability | \|  \| \| --- \| |
| Advising on drug and alcohol problems | \|  \| \| --- \| |
| Assessing the ageing worker and advising on work ability | \|  \| \| --- \| |
| Advising on vocational rehabilitation | \|  \| \| --- \| |
| Liaising with other health professionals in assessing capability for work | \|  \| \| --- \| |

**D Environmental Issues Related to Work Practice**

**Please RANK each subsection in terms of their importance;**

**1= of most importance to 6 = of least importance***

| Recognise and advise on health risks in the general environment arising from industrial activities | \|  \| \| --- \| |
| --- | --- | --- |
| Assess and advise on the control of environmental exposures from the workplace. | \|  \| \| --- \| |
| Understand when to obtain environmental monitoring | \|  \| \| --- \| |
| Interpret and explain the results of environmental monitoring. | \|  \| \| --- \| |
| Effectively communicate risk from various environmental exposures including water pollution, hazardous waste, sewage, household chemicals and radiation. | \|  \| \| --- \| |
| Identify and manage concerns about the health effects of human exposure to environmental hazards eg. toxins and pollutants. | \|  \| \| --- \| |

**E Health Promotion**

**Please RANK each subsection in terms of their importance;**

**1= of most importance to 3 = of least importance***

| Assessing needs for health promotion in a workforce. | \|  \| \| --- \| |
| --- | --- | --- |
| Give advice on nutritional and other healthy lifestyle issues. | \|  \| \| --- \| |
| Organising, providing and evaluating work related health promotion activities. | \|  \| \| --- \| |

**F Ethical and Legal issues**

**Please RANK each subsection in terms of their importance;**

**1= of most importance to 6 = of least importance***

| Be well-informed about acts, regulations, codes of practice and guidance relevant to the workplace setting. | \|  \| \| --- \| |
| --- | --- | --- |
| Providing advice to managers, safety representatives and employees of their legal obligations. | \|  \| \| --- \| |
| Evaluate compliance with new legislation | \|  \| \| --- \| |
| Responsibilities relating to data protection. | \|  \| \| --- \| |
| Respect the patient’s right to confidentiality. | \|  \| \| --- \| |
| Understand the process for gaining informed consent for clinical and research activities | \|  \| \| --- \| |

**G Clinical Governance/Clinical Improvement**

**Please RANK each subsection in terms of their importance;**

**1= of most importance to 6 = of least importance***

| Practise evidence based medicine. | \|  \| \| --- \| |
| --- | --- | --- |
| Be able to handle and deal with complaints in a focused and constructive manner. | \|  \| \| --- \| |
| Develop and institute clinical guidelines and integrated care pathways. Be aware of advantages and disadvantages of guidelines. | \|  \| \| --- \| |
| Report and investigate critical incidents. | \|  \| \| --- \| |
| Be able to balance risk and benefits with patients. | \|  \| \| --- \| |
| Be actively involved in clinical audit. | \|  \| \| --- \| |

**H Communication skills**

**Please RANK each subsection in terms of their importance;**

**1= of most importance to 9 = of least importance***

| Be able to communicate effectively both orally and in writing to patients and other stakeholders in a manner that they understand | \|  \| \| --- \| |
| --- | --- | --- |
| Prepare written reports on a range of topics for a range of groups including managers, unions (e.g. for safety representatives) and health professionals. | \|  \| \| --- \| |
| Demonstrate effective consultation skills. | \|  \| \| --- \| |
| Manage dissatisfied patients/ relatives. | \|  \| \| --- \| |
| Anticipate potential problems. | \|  \| \| --- \| |
| Be able to effectively participate in committees and to act as a chairperson. | \|  \| \| --- \| |
| Effective presentation skills. Make clear oral presentations to a range of audiences using audiovisual equipment. | \|  \| \| --- \| |
| Apply ethical principles when communicating with others about individuals. | \|  \| \| --- \| |
| Motivational interviewing skills | \|  \| \| --- \| |

**I Team working and leadership skills**

**Please RANK each subsection in terms of their importance;**

**1= of most importance to 6 = of least importance***

| Understand how a team works effectively | \|  \| \| --- \| |
| --- | --- | --- |
| Be an effective team player | \|  \| \| --- \| |
| Understand different leadership styles | \|  \| \| --- \| |
| Demonstrate leadership | \|  \| \| --- \| |
| Delegation | \|  \| \| --- \| |
| Influencing and negotiation | \|  \| \| --- \| |

**J Teaching & Educational Supervision**

**Please RANK each subsection in terms of their importance;**

**1= of most importance to 9 = of least importance***

| Identify learning outcomes and construct educational objectives. | \|  \| \| --- \| |
| --- | --- | --- |
| Design and deliver an effective teaching event or short course. | \|  \| \| --- \| |
| Teach large and small groups effectively. | \|  \| \| --- \| |
| Select and use appropriate teaching resources. | \|  \| \| --- \| |
| Give constructive effective feedback. | \|  \| \| --- \| |
| Evaluate programmes and events. | \|  \| \| --- \| |
| Use appropriate assessment methods. | \|  \| \| --- \| |
| Conduct effective appraisals. | \|  \| \| --- \| |
| Deliver effective mentorship | \|  \| \| --- \| |

**K Research methods**

**Please RANK each subsection in terms of their importance;**

**1= of most importance to 8 = of least importance***

| Be able to define a problem in terms of needs for an evidence base. | \|  \| \| --- \| |
| --- | --- | --- |
| Be able to undertake a literature search. | \|  \| \| --- \| |
| Be able to undertake a systematic and critical appraisal of scientific literature. | \|  \| \| --- \| |
| Be able to develop and execute an appropriate study design | \|  \| \| --- \| |
| Be able to use databases. | \|  \| \| --- \| |
| Be able to carry out basic statistical analyses. | \|  \| \| --- \| |
| Present investigation and results in the format of a research based report. | \|  \| \| --- \| |
| To understand the principles of research ethics and ethical considerations in research. | \|  \| \| --- \| |

**L Management skills**

**Please RANK each subsection in terms of their importance;**

**1= of most importance to 8= of least importance***

| Be able to understand the principles and practice of management. | \|  \| \| --- \| |
| --- | --- | --- |
| Be able to strategically plan and set objectives for delivering an occupational health service. | \|  \| \| --- \| |
| Finance management. Be able to negotiate and manage a budget/resources. | \|  \| \| --- \| |
| Evaluate the effectiveness and quality of an occupational health service. | \|  \| \| --- \| |
| Be able to market an occupational health service. | \|  \| \| --- \| |
| Define the roles and responsibilities of staff in providing an occupational health service | \|  \| \| --- \| |
| Support formulation of job descriptions. | \|  \| \| --- \| |
| Be able to manage a team. | \|  \| \| --- \| |

**Considering the above principle domain competencies and their subsections, we would ask you now to please RANK only the principle domains in order of importance using the scale 1 = of most importance and 12 = of least importance . Once again, no two items can be awarded the same score. ***

| **Good Clinical Care** | \|  \| \| --- \| |
| --- | --- | --- |
| **General principles of assessment & management of occupational hazards to health** | \|  \| \| --- \| |
| **Assessment of disability and fitness for work.** | \|  \| \| --- \| |
| **Environmental Issues Related to Work Practice** | \|  \| \| --- \| |
| **Health Promotion** | \|  \| \| --- \| |
| **Ethical and Legal issues** | \|  \| \| --- \| |
| **Clinical Governance/Clinical Improvement** | \|  \| \| --- \| |
| **Communication skills** | \|  \| \| --- \| |
| **Team working and leadership skills** | \|  \| \| --- \| |
| **Teaching & Educational Supervision** | \|  \| \| --- \| |
| **Research methods** | \|  \| \| --- \| |
| **Management skills** | \|  \| \| --- \| |

Table S2. Rank scores (mean, standardised mean,& weighted std rank) for all Principal domains and subsections

| **Principal Domain**  Subdomains | **Mean Rank Score** | **Standard Deviation** | **No of categories** | **Standardised Rank Score (StdRank)*** | **Weight** | **Weighted Standardise Rank (W_StdRank)**** |
| --- | --- | --- | --- | --- | --- | --- |
| **A. Good Clinical Care** | | | | | | |
| A1. Broad general medical knowledge of all common medical conditions including diagnostic criteria, evidence based treatment spectrum and prognosis | 3.8 | 2.1 | 9 | 4.1 | 1 | 4.1 |
| A2. Take and analyse a clinical and occupational history including an exposure history in a relevant, succint and systematic manner | 2.4 | 2.0 | 9 | 2.6 | 1 | 2.6 |
| A3. Perform a reliable and appropriate examination | 5.8 | 2.4 | 9 | 6.4 | 1 | 6.4 |
| A4. Interpret the results of investigations, including especially those relating to occupational attribution and functional prognosis | 5.4 | 1.9 | 9 | 5.9 | 1 | 5.9 |
| A5. Perform investigations competently where relevant | 6.4 | 1.9 | 9 | 7.0 | 1 | 7.0 |
| A6. Record concisely, accurately, confidentially and legibly all medical records, and date and sign all records | 3.8 | 2.0 | 9 | 4.2 | 1 | 4.2 |
| A7. Show empathy with and listen to employee. | 5.3 | 2.6 | 9 | 5.8 | 1 | 5.8 |
| A8. Appreciate the importance and interaction of psychological and social factors in patient’s disease and illness behaviour. | 5.1 | 2.2 | 9 | 5.6 | 1 | 5.6 |
| A9. Manage time and problems effectively. | 7.1 | 2.6 | 9 | 7.8 | 1 | 7.8 |
| **B. General principles of assessment & management of occupational hazards to health** | | | | | | |
| B1. Understand and apply the principles of risk assessment- ie recognition of potential hazards in the work environment, evaluating risks and providing advice and information on control measures. | 2.2 | 1.9 | 11 | 2.1 | 2 | 4.2 |
| B2. Provide advice on medical aspects/ factors relevant to the risk assessment. | 3.2 | 1.7 | 11 | 3.0 | 2 | 5.9 |
| B3. Carry out a workplace visit and produce a report. | 5.1 | 2.1 | 11 | 4.7 | 2 | 9.3 |
| B4. Understand the core principles of occupational/ industrial hygiene and be able to interpret reports and related measurements. | 5.6 | 2.1 | 11 | 5.1 | 2 | 10.3 |
| B5. Understand the core principles of ergonomics and be able to interpret reports. | 6.1 | 1.5 | 11 | 5.6 | 2 | 11.3 |
| B6. Understand the core principles of toxicology and be able to interpret reports and related measurements. | 7.8 | 1.7 | 11 | 7.2 | 2 | 14.3 |
| B7. Identify work related ill health and provide advice on prognosis, prevention and management. | 3.4 | 2.4 | 11 | 3.2 | 2 | 6.3 |
| B8. Carry out and evaluate health surveillance including biological monitoring for workers exposed to occupational hazards. | 4.6 | 2.2 | 11 | 4.2 | 2 | 8.4 |
| B9. Evaluate and advise on first aid facilities in the workplace. | 9.1 | 1.5 | 11 | 8.3 | 2 | 16.7 |
| B10. Advising on disaster and contingency planning within the organisation. | 9.3 | 1.8 | 11 | 8.5 | 2 | 17.0 |
| B11. Understand the principles of Travel Medicine | 9.6 | 2.3 | 11 | 8.7 | 2 | 17.4 |
| **C. Assessment of disability and fitness for work.** | | | | | | |
| C1. Assessing and advising on impairment, disability and fitness for work | 1.6 | 0.9 | 10 | 1.6 | 4 | 6.4 |
| C2. Advising on rehabilitation | 3.4 | 1.6 | 10 | 3.4 | 4 | 13.7 |
| C3. Advising on redeployment | 5.3 | 1.8 | 10 | 5.3 | 4 | 21.1 |
| C4. Assessing and advising on early retirement due to ill-health | 7.1 | 2.2 | 10 | 7.1 | 4 | 28.2 |
| C5. Assessing and advising on sickness absence | 3.3 | 2.5 | 10 | 3.3 | 4 | 13.1 |
| C6. Advising on legal issues related to disability | 5.8 | 2.5 | 10 | 5.8 | 4 | 23.3 |
| C7. Advising on drug and alcohol problems | 7.8 | 1.6 | 10 | 7.8 | 4 | 31.0 |
| C8. Assessing the ageing worker and advising on work ability | 7.0 | 2.2 | 10 | 7.0 | 4 | 27.9 |
| C9. Advising on vocational rehabilitation | 7.5 | 2.3 | 10 | 7.5 | 4 | 30.1 |
| C10. Liaising with other health professionals in assessing capability for work | 6.3 | 2.8 | 10 | 6.3 | 4 | 25.1 |
| **D. Environmental Issues Related to Work Practice** | | | | | | |
| D1. Recognise and advise on health risks in the general environment arising from industrial activities. | 1.7 | 1.2 | 6 | 2.3 | 8 | 18.3 |
| D2. Assess and advise on the control of environmental exposures from the workplace. | 3.1 | 1.3 | 6 | 4.7 | 8 | 37.8 |
| D3. Understand when to obtain environmental monitoring. | 3.6 | 1.1 | 6 | 5.7 | 8 | 45.8 |
| D4. Interpret and explain the results of environmental monitoring. | 4.8 | 1.1 | 6 | 7.8 | 8 | 62.3 |
| D5. Effectively communicate risk from various environmental exposures including water pollution, hazardous waste, sewage, household chemicals and radiation. | 4.1 | 1.6 | 6 | 6.6 | 8 | 53.0 |
| D6. Identify and manage concerns about the health effects of human exposure to environmental hazards eg. toxins and pollutants. | 3.7 | 2.0 | 6 | 5.9 | 8 | 46.8 |
| **E. Health Promotion** | | | | | | |
| E1. Assessing needs for health promotion in a workforce. | 1.3 | 0.5 | 3 | 2.5 | 10 | 25.3 |
| E2. Give advice on nutritional and other healthy lifestyle issues. | 2.8 | 0.5 | 3 | 8.9 | 10 | 88.8 |
| E3. Organising, providing and evaluating work related health promotion activities. | 1.9 | 0.7 | 3 | 5.1 | 10 | 51.0 |
| **F. Ethical and Legal issues** | | | | | | |
| F1. Be well-informed about acts, regulations, codes of practice and guidance relevant to the workplace setting. | 2.1 | 1.2 | 6 | 2.9 | 6 | 17.6 |
| F2. Providing advice to managers, safety representatives and employees of their legal obligations. | 3.8 | 1.2 | 6 | 6.1 | 6 | 36.7 |
| F3. Evaluate compliance with new legislation | 4.7 | 1.3 | 6 | 7.6 | 6 | 45.7 |
| F4. Responsibilities relating to data protection. | 3.8 | 1.4 | 6 | 6.0 | 6 | 36.3 |
| F5. Respect the patient’s right to confidentiality. | 2.1 | 1.3 | 6 | 3.0 | 6 | 18.2 |
| F6. Understand the process for gaining informed consent for clinical and research activities | 4.5 | 1.7 | 6 | 7.3 | 6 | 43.6 |
| **G. Clinical Governance/Clinical Improvement** | | | | | | |
| G1. Practise evidence based medicine. | 1.4 | 0.9 | 6 | 1.7 | 5 | 8.4 |
| G2. Be able to handle and deal with complaints in a focused and constructive manner. | 4.2 | 1.2 | 6 | 6.7 | 5 | 33.6 |
| G3. Develop and institute clinical guidelines and integrated care pathways. Be aware of advantages and disadvantages of guidelines. | 3.1 | 1.5 | 6 | 4.7 | 5 | 23.6 |
| G4. Report and investigate critical incidents. | 4.4 | 1.3 | 6 | 7.0 | 5 | 35.2 |
| G5. Be able to balance risk and benefits with patients. | 3.9 | 1.6 | 6 | 6.3 | 5 | 31.5 |
| G6. Be actively involved in clinical audit. | 4.1 | 1.6 | 6 | 6.5 | 5 | 32.6 |
| **H. Communication skills** | | | | | | |
| H1. Be able to communicate effectively both orally and in writing to patients and other stakeholders in a manner that they understand | 1.4 | 0.6 | 9 | 1.4 | 3 | 4.2 |
| H2. Prepare written reports on a range of topics for a range of groups including managers, unions (e.g. for safety representatives) and health professionals. | 3.2 | 1.6 | 9 | 3.5 | 3 | 10.5 |
| H3. Demonstrate effective consultation skills. | 3.2 | 1.6 | 9 | 3.4 | 3 | 10.3 |
| H4. Manage dissatisfied patients/ relatives. | 6.0 | 1.6 | 9 | 6.7 | 3 | 20.0 |
| H5. Anticipate potential problems. | 5.2 | 1.5 | 9 | 5.7 | 3 | 17.0 |
| H6. Be able to effectively participate in committees and to act as a chairperson. | 7.3 | 1.3 | 9 | 8.1 | 3 | 24.2 |
| H7. Effective presentation skills. Make clear oral presentations to a range of audiences using audiovisual equipment. | 7.0 | 1.5 | 9 | 7.8 | 3 | 23.3 |
| H8. Apply ethical principles when communicating with others about individuals. | 4.3 | 2.4 | 9 | 4.8 | 3 | 14.3 |
| H9. Motivational interviewing skills | 7.4 | 1.9 | 9 | 8.3 | 3 | 24.8 |
| **I. Team working and leadership skills** | | | | | | |
| I1. Understand how a team works effectively | 2.2 | 1.4 | 6 | 3.1 | 7 | 21.9 |
| I2. Be an effective team player | 3.0 | 1.6 | 6 | 4.6 | 7 | 32.0 |
| I3. Understand different leadership styles | 3.4 | 1.5 | 6 | 5.4 | 7 | 37.8 |
| I4. Demonstrate leadership | 3.2 | 1.5 | 6 | 5.0 | 7 | 34.7 |
| I5. Delegation | 5.1 | 1.0 | 6 | 8.4 | 7 | 58.5 |
| I6. Influencing and negotiation | 4.1 | 1.6 | 6 | 6.6 | 7 | 46.2 |
| **J. Teaching & Educational Supervision** | | | | | | |
| J1. Identify learning outcomes and construct educational objectives. | 2.1 | 1.6 | 9 | 2.3 | 11 | 24.9 |
| J2. Design and deliver an effective teaching event or short course. | 4.8 | 2.6 | 9 | 5.3 | 11 | 58.3 |
| J3.Teach large and small groups effectively. | 5.6 | 2.2 | 9 | 6.1 | 11 | 67.4 |
| J4. Select and use appropriate teaching resources. | 5.0 | 2.0 | 9 | 5.5 | 11 | 60.3 |
| J5. Give constructive effective feedback. | 4.7 | 2.0 | 9 | 5.1 | 11 | 56.5 |
| J6. Evaluate programmes and events. | 6.5 | 1.8 | 9 | 7.2 | 11 | 79.3 |
| J7. Use appropriate assessment methods. | 5.6 | 2.3 | 9 | 6.1 | 11 | 67.6 |
| J8. Conduct effective appraisals. | 5.4 | 2.8 | 9 | 6.0 | 11 | 65.6 |
| J9. Deliver effective mentorship | 5.3 | 3.2 | 9 | 5.9 | 11 | 64.7 |
| **K. Research methods** | | | | | | |
| K1. Be able to define a problem in terms of needs for an evidence base. | 1.9 | 1.3 | 8 | 2.1 | 12 | 25.5 |
| K2. Be able to undertake a literature search. | 3.2 | 1.3 | 8 | 3.8 | 12 | 46.2 |
| K3. Be able to undertake a systematic and critical appraisal of scientific literature. | 3.8 | 1.4 | 8 | 4.7 | 12 | 55.8 |
| K4. Be able to develop and execute an appropriate study design | 5.0 | 1.6 | 8 | 6.2 | 12 | 74.0 |
| K5. Be able to use databases. | 4.7 | 2.1 | 8 | 5.8 | 12 | 69.3 |
| K6. Be able to carry out basic statistical analyses. | 6.1 | 1.5 | 8 | 7.5 | 12 | 90.2 |
| K7. Present investigation and results in the format of a research based report. | 6.9 | 1.4 | 8 | 8.6 | 12 | 103.5 |
| K8. To understand the principles of research ethics and ethical considerations in research. | 4.3 | 2.9 | 8 | 5.3 | 12 | 63.5 |
| **L. Management skills** | | | | | | |
| L1. Be able to understand the principles and practice of management. | 2.5 | 1.5 | 8 | 3.0 | 9 | 26.6 |
| L2. Be able to strategically plan and set objectives for delivering an occupational health service. | 2.6 | 1.6 | 8 | 3.0 | 9 | 27.2 |
| L3. Finance management. Be able to negotiate and manage a budget/resources. | 5.1 | 2.0 | 8 | 6.3 | 9 | 56.3 |
| L4. Evaluate the effectiveness and quality of an occupational health service. | 4.1 | 1.6 | 8 | 4.9 | 9 | 44.5 |
| L5. Be able to market an occupational health service. | 6.2 | 1.7 | 8 | 7.7 | 9 | 69.1 |
| L6. Define the roles and responsibilities of staff in providing an occupational health service | 4.4 | 1.9 | 8 | 5.4 | 9 | 48.7 |
| L7. Support formulation of job descriptions. | 6.8 | 1.6 | 8 | 8.5 | 9 | 76.4 |
| L8. Be able to manage a team. | 4.3 | 2.2 | 8 | 5.2 | 9 | 47.2 |
|  |  |  |  |  |  |  |
| **M. Principal Domains** | | | | | | |
| M1. Good Clinical Care | 2.6 | 2.5 | 12 | 2.3 |  |  |
| M2. General principles of assessment & management of occupational hazards to health | 3.2 | 2.3 | 12 | 2.8 |  |  |
| M3. Assessment of disability and fitness for work. | 4.6 | 2.1 | 12 | 3.9 |  |  |
| M4. Environmental Issues Related to Work Practice | 7.4 | 2.1 | 12 | 6.2 |  |  |
| M5. Health Promotion | 8.7 | 2.4 | 12 | 7.3 |  |  |
| M6. Ethical and Legal issues | 5.8 | 2.1 | 12 | 4.9 |  |  |
| M7. Clinical Governance/Clinical Improvement | 5.8 | 2.6 | 12 | 4.9 |  |  |
| M8. Communication skills | 4.2 | 2.7 | 12 | 3.6 |  |  |
| M9. Team working and leadership skills | 6.4 | 2.6 | 12 | 5.4 |  |  |
| M10.Teaching & Educational Supervision | 9.9 | 1.6 | 12 | 8.3 |  |  |
| M11. Research methods | 11.3 | 1.2 | 12 | 9.4 |  |  |
| M12. Management skills | 8.3 | 3.1 | 12 | 7.0 |  |  |

*Standardised Ranks (StdRank) Score to a scale of 1-10 using the formula: *9*((Average Rank-1)/(No. of categories-1))+1*

** Weighted Standardise Rank (W_StdRank) = Weight of ranked Principal Domain * StdRank
